# A New Method of Delivering the After-Coming Head in Contracted Pelvis

**Published:** 1871

**Authors:** William Goodell


ARTICLE 217.—*A New Method of Delivering the After-coming Head in Contracted Pelvis.* By WILLIAM GOODELL, M. D.

[American Journal of Obstetrics, November, 1870.]

Dr. Goodell, in a communication to the Philadelphia Obstetrical Society, gives the following account of this method, which he has successfully employed:

"In a brim contracted in its antero-posterior diameter, the sacral side of the after-coming head becomes fixed on the point of the projecting promontory, and its extrication can take place only when the pubic side of the head can be made to revolve around the promontory as a centre of motion, by descending over the smooth suface of the symphysis. And this can the more readily be done in such cases, because the sacrum is so scooped out and so shortened that traction can be often made in a line posterior to the axis of the superior strait. Bearing this fact in mind, after grasping the neck of the child with one hand, and the ankles with the other, the physician should make his first movement of traction in the axis of the outlet—indeed, in a line anterior to this axis—by at the same time forcing the child's neck against the pubes, for then the pubic side of the head will recede from the inlet, whilst the sacral side will proportionately descend over the point of the promontory, and affront the inlet. This change of position can be materially aided by an intelligent assistant, who will make pressure with both hands, through the flaccid abdominal walls, upon the vault of the child's head, from before, backwards and downwards. By this manœuvre the promontory will nip the head at a point higher up, and nearer to its vault; hence the arm of the lever, measured by a line drawn from the base of the skull to the point nipped by the promontory, will be correspondingly lengthened, an advantage not to be overlooked. If now, without for a moment relaxing the original traction-force, its direction be reversed, and the body of the child be swept backwards upon the coccyx—the neck also being forced downwards and backwards into the hollow of the saccrum—and if the assistant at the same time reverses the direction of the external pressure upon the vault of the head, making it now from behind, forwards and downwards, the pubic side of the child's head is made to revolve around the promontory and descend with the least expenditure of traction force, and consequently with a much greater chance of safety to the child. Of course, as soon as the head has passed the brim, the line of traction must be changed to that of Carus' curve. Dr. G. stated that, by this method, he had thus far delivered three women with unusually bad pelves; in one of them the child's head had to be lessened in a previous labor. His last case presented so many points of interest, he would briefly relate it: A secundipara, in her previous labor, had fortunately been prematurely delivered at the end of the seventh month of gestation. The child was still; weighed under 5 lbs., and yet her labor lasted over forty-eight hours, six of them being attended with extreme suffering and violent expulsive pains. On the present occasion she went to term against the wishes of her attending physician, who had urged the induction of premature labor. At eight, A.M., when labor had lasted ten hours, Dr. G. found the water dribbling away from an expanded os uteri. The head, presenting the occiput to the left ilium, would not bear upon the cervix, but rolled about upon the shelf formed by the promontory. By careful measurement with the finger, the conjugate diameter of the brim was estimated at a trifle over three inches. It was his intention to dilate the os with the water-bags, and apply the forceps. But while he was preparing to do so, the woman suddenly began to twitch her muscles, start convulsively, and to complain of blindness and intense headache. Fearing an attack of eclampsia, he determined to deliver at once. The woman was accordingly thoroughly etherized, and brought down to the edge of the bed in the dorsal decubitus. Two fingers of the left hand were then squeezed into the os, and ultimately a third, by which, in conjunction with the right hand externally, version was made with great ease. The foot having been drawn down, a short time was allowed to elapse before the body and arms were delivered, in order that the breech might dilate the os. Then, by following the method he had just described, the child was soon delivered in an asphyxiated condition, but promptly recovered. It presented a deep indentation on the right side of its head, weighed 7lbs. 12ozs., and next day measured 3½ inches at the bi-parietal diameter. In conclusion, Dr. G. remarked that so much power and time were gained in ordinary pelvic presentations by the plan of simply pushing the head through the brim, a plan which he at first learnt from Prof. R. A. F. Penrose, that he was surprised it was not oftener resorted to. For although the neck of the child could bear a great strain without breaking, it was desirable to diminish the chances of this risk, as well as to deliver as soon as possible, since few children survived a delay of the brim of over five minutes' duration."—*Half-Yearly Abstract.*

